# Effect of Initial Aluminum Alloy Particle Size on the Damage of Carbon Nanotubes during Ball Milling

**DOI:** 10.3390/ma9030173

**Published:** 2016-03-08

**Authors:** Xian Zhu, Yu Guang Zhao, Min Wu, Hui Yuan Wang, Qi Chuan Jiang

**Affiliations:** Key Laboratory of Automobile Materials of Ministry of Education, School of Materials Science and Engineering, Jilin University, Changchun 130025, China; zhuxian43060701@163.com (X.Z.); zhaoyg@jlu.edu.cn (Y.G.Z.); wuminwm2015@163.com (M.W.); jiangqc@jlu.edu.cn (Q.C.J.)

**Keywords:** metal matrix composites, carbon nanotube, Raman spectroscopy, aluminum alloy, ball milling

## Abstract

Damage to carbon nanotubes (CNTs) during the fabrication process of CNT reinforced composites has great influence on their mechanical properties. In this study, the 2014 Al with powder sizes of 20, 9 and 5 μm was selected to study the effect of initial particle size on the damage to carbon nanotubes (CNTs) during ball milling. The result shows that for CNTs in the ball milled CNT/Al (with powder size of 20 and 9 μm) mixtures, the intensity ratio of the D band and the G band (ID/IG) first increased and then reached a plateau, mainly because most of the CNTs are embedded, to a certain extent, in the aluminum powder after milling, which could protect the CNTs from damage during further milling. While for CNTs in the ball milled CNT/Al (with powder size of 5 μm) mixture, the ID/IG ratio continues to climb from 1.31 to 2.33 with time, indicating continuous damage to the CNTs occurs during the milling. Differential scanning calorimetry (DSC) analysis demonstrates that the chemical instability increased with an increase in the damage level of CNTs, resulting in the formation of aluminum carbide (Al_4_C_3_) at a lower temperature before the melting of aluminum, which is detrimental to their mechanical properties.

## 1. Introduction

Since discovered by Iijima [1], carbon nanotubes (CNTs) are considered to be ideal reinforcements of composites due to their exceptional strength, high Young’s modulus and low density [2,3]. Among many candidate matrix materials for lightweight high-strength composites, aluminum alloys have been considered to be preferential due to their relatively low density and reasonable mechanical properties. Recently, aluminum matrix composites have been receiving more attention in aerospace, military and automobiles, due to their high tensile strength, hardness and modulus, as well as their high wear resistance compared to the matrix [4,5,6,7,8,9,10].

In the process of manufacturing the Al-CNT composites, dispersion of CNTs is a vital step, and is still challenging due to the high aspect ratio and strong van der Waal’s force attraction among the CNTs, particularly the ones that are supplied in the form of entangled bundles [11,12,13]. Ball milling, also called mechanical alloying, has been proven effective to uniformly disperse CNTs within the aluminum matrix [14]. However, the repeated deformation, cold welding and fragmentation of particles during the ball milling process would lead to a morphology change in the CNTs and could even cause total destruction if harsh milling conditions are utilized. The CNTs can be transformed into short and open-tipped nanotubes because of locally generated high pressure from the collisions between balls in the milling chamber [15,16,17,18]. In addition, the mechanical alloying may result in amorphous as well as disrupted tubular structures [12,19,20,21]. In the contrary, some other studies have claimed that cold welding of the aluminum matrix particles around the CNTs protects them from damage [22,23].

The damage of CNTs has an intimate relationship to the mechanical properties of the composites. From the research of Ci *et al.* [24], carbide nanostructures were preferentially formed at the sites of structural disorder, nano-defects, and the open ends of multi-walled carbon nanotubes (MWCNTs). In addition, Nayan *et al.* [25] reported that the reaction between damaged CNTs and Al could occur at a lower temperature during powder metallurgy processing. The formation of the Al_4_C_3_ phase has a significant influence on the mechanical properties of the composites. Although a number of studies on the CNT damage during the ball milling process have been reported such as ball milling time [17], ball milling intensity [16] and the ball milling atmosphere [26,27], the effect of aluminum alloy powder size on the damage of CNTs has not been investigated yet.

In this study, three size levels of initial aluminum alloy powder were ball milled with 2.0 wt % CNTs under different ball mill conditions. The effect of the initial particle size on the damage of the CNT was meticulously evaluated. Also, CNTs reinforced 2014 Al alloy composites were fabricated by a hot pressing process to characterize their mechanical properties.

## 2. Experimental Section

**Figure 1:**
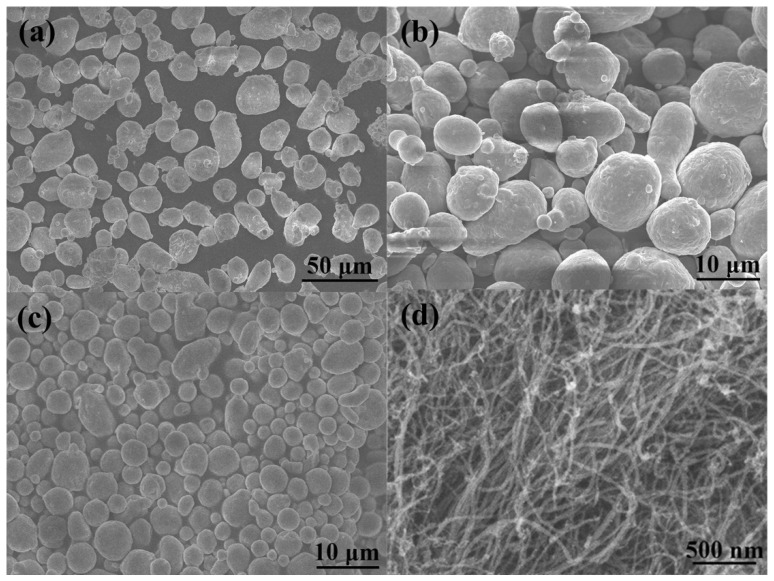

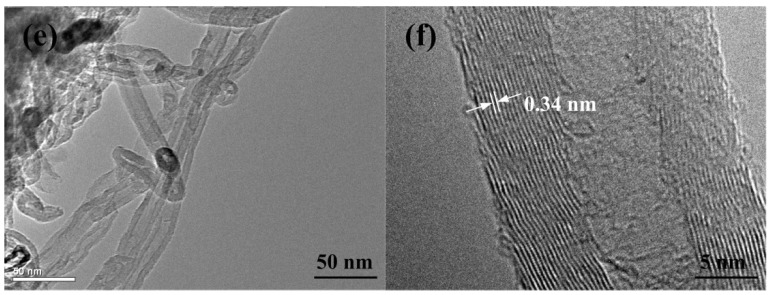
Morphologies of the as-received powders. FESEM micrographs of the 2014 aluminum alloy powders with different particle size: (**a**) 20 μm; (**b**) 9 μm; (**c**) 5 μm; Micrographs of carbon nanotubes: (**d**) FESEM; (**e**) TEM; (**f**) HRTEM.

Three kinds of commercially available gas atomized spherical 2014 aluminum alloy powders with powder sizes of 20, 9 and 5 μm were used as the matrix powder (Figure 1a–c). Chemical composition of the 2014 Al is listed in Table 1. CNTs with an average diameter of 20–30 nm and a length of 10–30 μm were selected as the reinforcements. The CNTs, which were synthesized by the chemical vapor deposition (CVD) method, are present as entangled bundles due to the highly specific aspect ratio and strong van der Waal’s force attraction between them (Figure 1d). From Transmission Electron Microscopy (TEM) observation, the multi-walled CNTs were seen to exhibit a herringbone structure (Figure 1e), and the distance between adjacent walls was about 0.34 nm (Figure 1f). 

The mixture of Al (with powder size of 20 μm) and 2.0 wt % CNTs with a total weight of 40 g was placed in a 250 mL stainless steel jar containing 100 ball bearing steels 10 mm in diameter, which gave a ball to powder weight ratio (BPR) of 10:1. No process control agent was added. Then, the jar was sealed and agitated at a constant speed of 200 rpm on a planetary ball mill. Selected milling times were 3, 6, 12, 18 and 24 h, respectively. Such ball-milling conditions, *viz.* the BPR, the rotating speed and the concentration of CNTs, are identical to those employed by Esawi and Morsi [23]. The mixtures of Al (with powder size of 9 μm) and 2.0 wt % CNTs, and Al (with powder size of 5 μm) and 2.0 wt % CNTs were subjected to the same ball milling process. Finally, the ball-milled mixture powders were packed and cold compacted at a pressure of 50 MPa for 2 min followed by hot sintering for 50 min at 793 K and pressing at a pressure of 50 MPa for 10 min in a self-made vessel in vacuum, respectively. The sintered composites were furnace cooled to room temperature. Then, they were solution treated at 775 K for 2 h and naturally aged for more than 96 h before test.

Morphologies of the ball-milled powder were characterized by a field emission scanning electron microscopy (FESEM) (JSM-6700F, JEOL, Tokyo, Japan) and transmission electron microscope (TEM) (fei F20, Philips, Amsterdam, The Netherlands). The size distribution of the milled powders was analyzed using image analysis software (Nano measure). Raman spectroscopy of the composite powder was obtained by REINSHAW in Via Raman spectroscope (Reinshaw, London, UK) with an excitation laser wavelength of 633 nm in the range of 1000–2000 cm^−1^. Phase constituent of the sintered composites were characterized by X-ray diffraction (XRD) (D/Max 2500PC, Rigaku, Tokyo, Japan) using Cu Kα radiation in the range of 20°–80° with a scanning speed of 4°/min and an acquisition step of 0.02° (2θ). The DSC experiments were conducted by using the Model Rigaku-8510 (Rigaku, Tokyo, Japan) apparatus at a scan rate of 10 °C/min under the flow of high purity argon. Micro-hardness tests were conducted on a 1600–5122VD MICROMET 5104 (Buehler, Chicago, IL, USA) using a load of 25 g and a dwell time of 15 s. The reported value was the average of at least 5 indentations. The density of all samples was measured by Archimedes’ method. The porosities were calculated by the equation:

(1) $$p=1-\rho_{e}/\rho_{t}$$

where ρ_e_ is the experiment density, and ρ_t_ is the theoretical density of the composites. The density of 2.8 g·cm^−3^ for the matrix and 1.7 g·cm^−3^ [28] for the CNTs were used to calculate theoretical density of the composites. Cylindrical specimens of CNT/Al2014 composites with length-to-diameter ratio of 2:1 (with the length and diameter of 6mm and 3 mm, respectively) were prepared from the sintered composites and used for compression tests. Uniaxial compression experiments at strain rate of 3 × 10^−4^ mm/s were performed using an Instron-type machine (INSTRON 5869, Instron Corporation, Cambridge, MA, USA) at room temperature.

## 3. Results and Discussion

**Figure 2:**
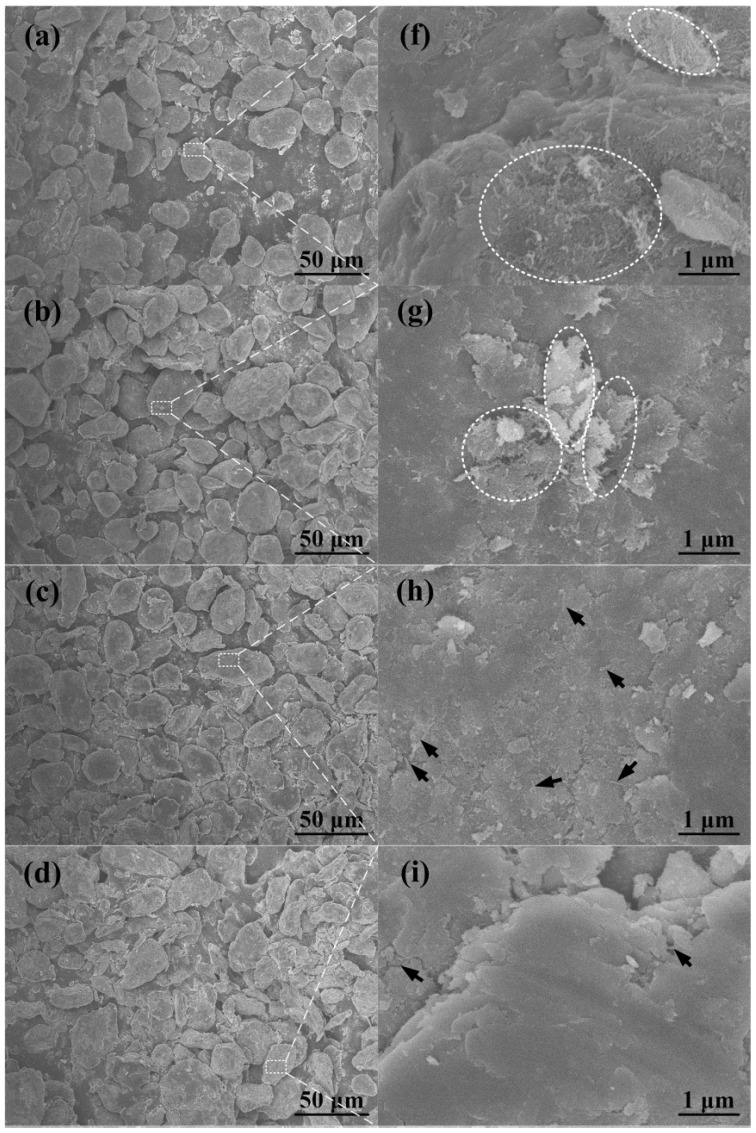

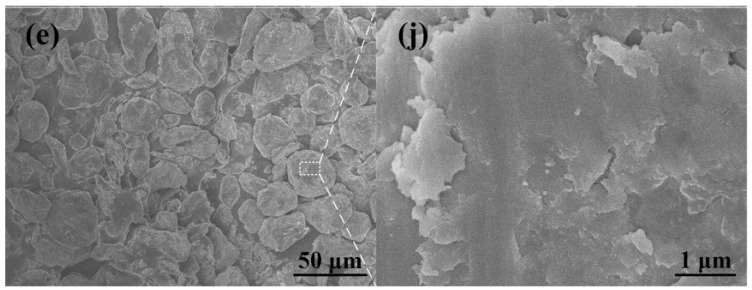
FESEM micrographs of CNT/Al (with powder size of 20 μm) mixture ball milled for (**a**) 3 h, (**b**) 6 h, (**c**) 12 h, (**d**) 18 h and (**e**) 24 h. From (**f**) to (**j**) are the magnified images of the white dotted regions in (**a**–**e**), respectively.

Figure 2a–e shows FESEM images of the 2.0 wt % CNTs/Al (with powder size of 20 μm) mixture after different ball milling times varying from 6 h to 24 h, and Figure 2f–i is the magnified images of the white dotted boxes in Figure 2a–e, respectively. The clusters of CNTs were bounded by white dotted elliptical rings, and the tips of the CNTs embedded in the matrix were marked by black arrows. Note that the ductile aluminum powder becomes flattened by the ball-powder-ball collisions, and some bright particles with a size of about several microns are observed on the surface of the flattened aluminum powder, after the ball milling times of 3 h and 6 h. The bright particles in the magnified image of Figure 2f,g are identified to be constituted CNT bundles, as indicated by the black arrows. In this stage, these bundles would float on the powder surface. When the ball milling time increases to 12 h, these are not observed any longer (Figure 2c). CNTs seem to be dispersed on the surface of the flattened aluminum powder (Figure 2h). With further increases in the ball milling time to 18 h, some of the aluminum powders gradually become cold-welded to each other (Figure 2d). CNTs are mostly embedded in the aluminum powder and are rarely observed on the surface of the aluminum powder (Figure 2i). After 24 h of milling, no CNTs are observed on the surface (Figure 2e,j).

**Figure 3:**
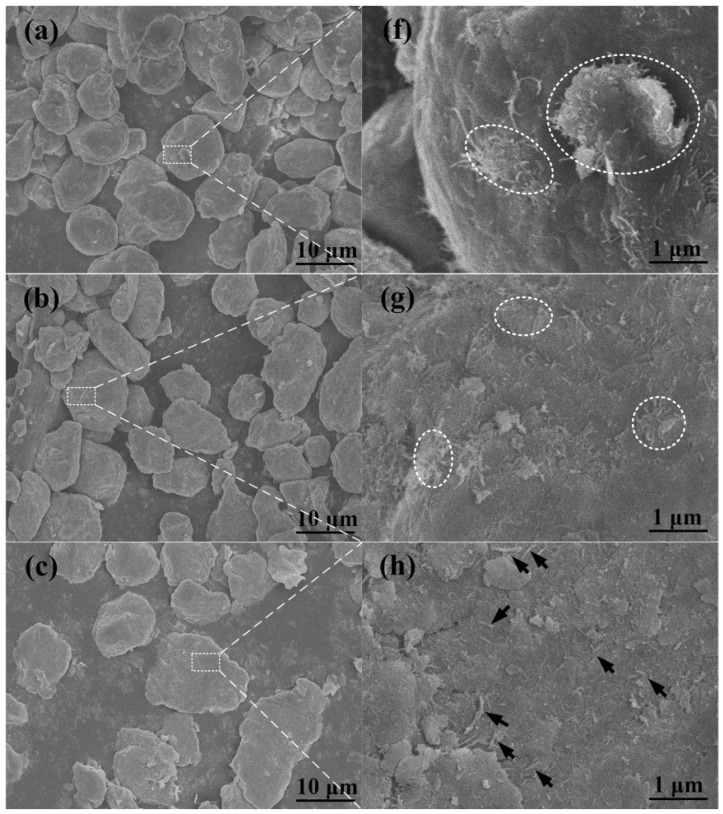

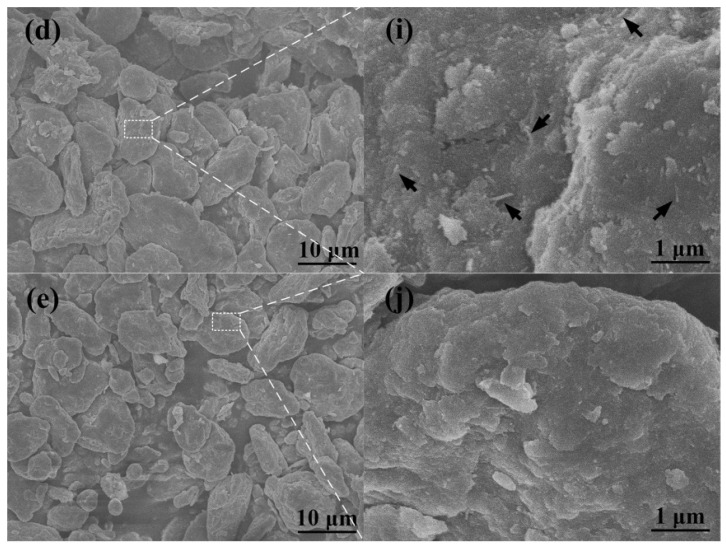
FESEM micrographs of CNT/Al (with powder size of 9 μm) mixture ball milled for (**a**) 3 h; (**b**) 6 h; (**c**) 12 h; (**d**) 18 h; (**e**) 24 h. The magnified images of the white dotted regions for (**f**) 3 h; (**g**) 6 h; (**h**) 12 h; (**i**) 18h; (**j**) 24 h.

The morphologies of the 2.0 wt % CNTs/Al (with powder size of 9 μm) mixture after different ball milling times varying from 3 h to 24 h are shown in Figure 3a–e, and Figure 3f–j is the magnified images of the white dotted boxes in Figure 3a–e, respectively. The clusters of CNTs were bounded by white dotted elliptical rings, and the tips of the CNTs embedded in the matrix were marked by black arrows. As can be seen, the clusters of CNTs on the flattened aluminum powders become smaller than 1 μm (Figure 3b,g). With further milling of 12 h, the aluminum gets more flattened, clusters of CNTs on the aluminum powder surface disappear and tips of CNTs are observed as indicated by black arrows in Figure 3h. As the milling proceeds for 18 h, the large flattened aluminum powder becomes fractured and a few of the CNTs are observed on the surface of the aluminum powder (Figure 3d,i). After 24 h of milling, the fractured pieces again become cold-welded to each other, and become rounded to some extent (Figure 3e). Additionally, the CNTs seem to be dispersed and are located deeper inside the powder through plastic deformation of the matrix, as shown in Figure 3j.

**Figure 4:**
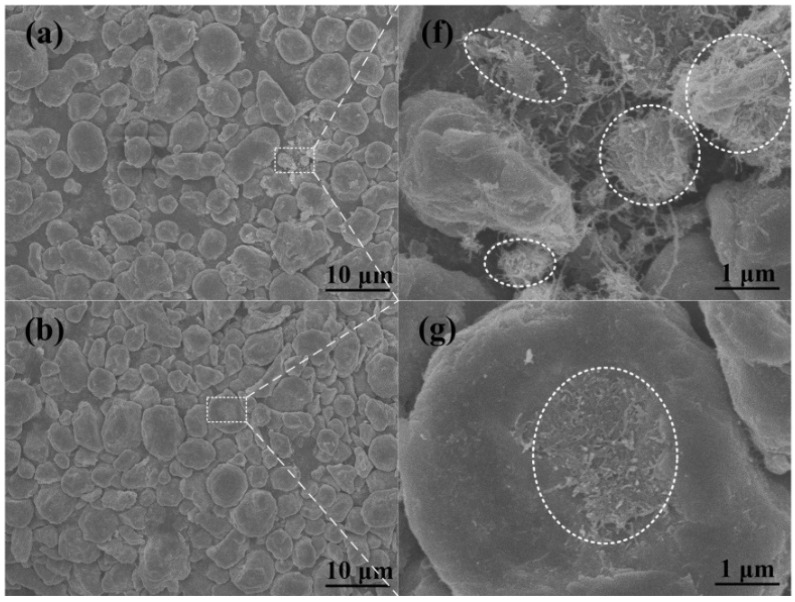

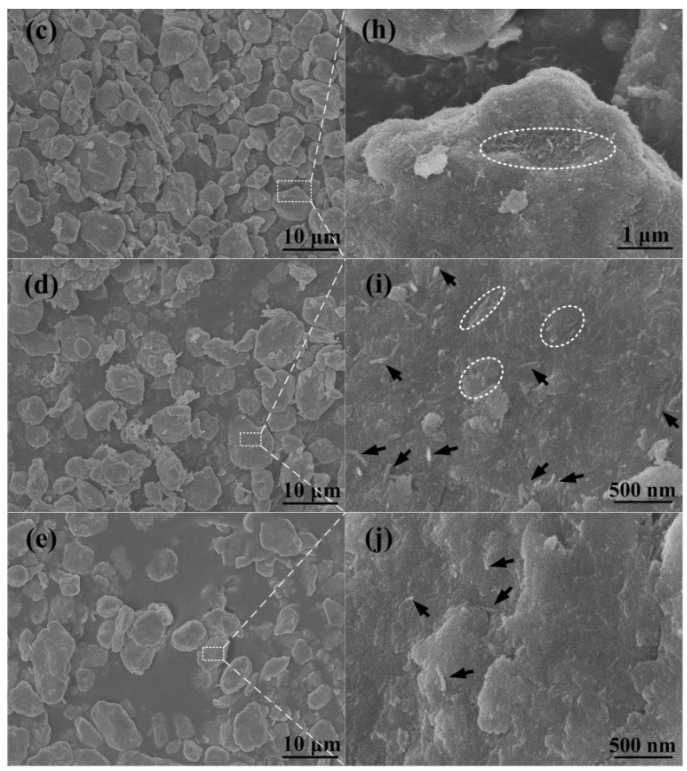
FESEM micrographs of CNT/Al (with powder size of 5 μm) mixture ball milled for (**a**) 3 h, (**b**) 6 h, (**c**) 12 h, (**d**) 18 h and (**e**) 24 h. From (**f**) to (**j**) are the magnified images of the white dotted regions in (**a**–**e**), respectively.

Figure 4a–e presents the FESEM images of the 2.0 wt % CNTs/Al (with powder size of 5 μm) mixture after different ball milling times varying from 3 h to 24 h, and Figure 4f–j is the magnified images of the white dotted boxes in Figure 4a–e, respectively. The clusters of CNTs were bounded by white dotted elliptical rings, and the tips of the CNTs embedded in the matrix were marked by black arrows. It is noticeable that the CNT clusters exsit on the surface of the aluminum powder during the milling time from 3 to 18 h (Figure 4f–i). Further increasing the milling time to 24 h, the CNT clusters are hardly observed and a few of the CNTs are found to be dispersed on the aluminum powder (Figure 4h). Simutaneously, the aluminum powder is deformed into a flaky shape and becomes fractured into smaller pieces after 12 h of milling (Figure 4a–c). Then, the flaky aluminum powder starts to become cold-welded to each other after 18 h and 24 h of milling, respectively (Figure 4d,e). Accordingly, it seems that CNTs need more time to disperse into the aluminum powder with a powder size of 5 μm.

These observations demonstrate that the particle size of the aluminum powder and the milling time have a intimate relationship with the morphologies of the ball-milled aluminum powder and the dispersion state of CNTs in the aluminum matrix. The larger the initial aluminum alloy powder, the easier entrappment of the carbon nanotubes during ball milling.

**Figure 5:**
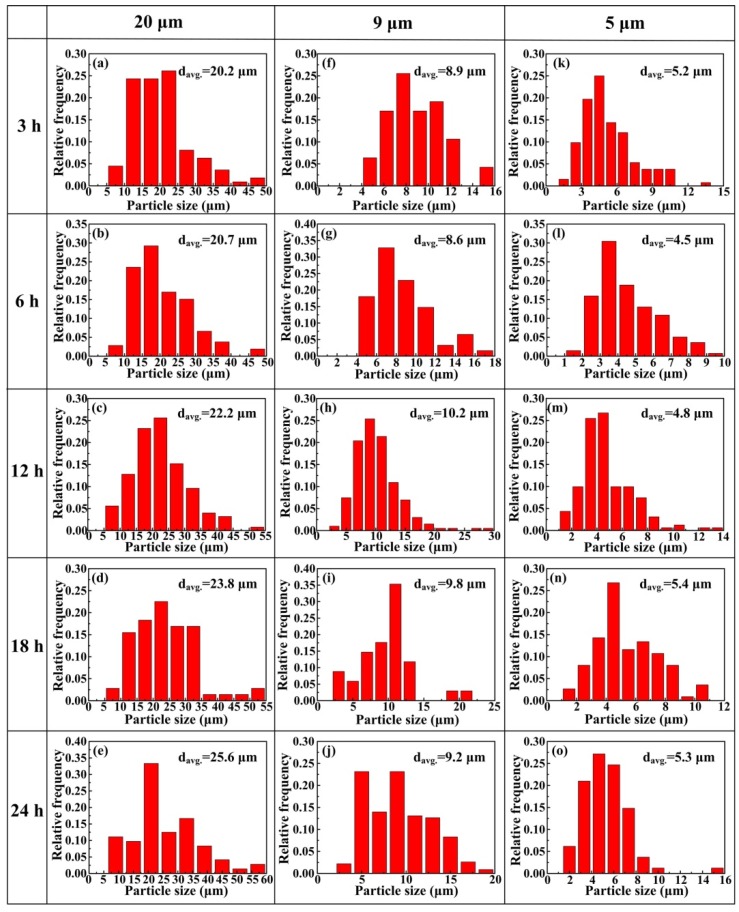
Histogram of the size distribution of the CNT/Al composites: (**a**) to (**e**), (**f**) to (**j**) and (**k**) to (**o**) are for the CNT/Al (with the Al powder sizes of 20, 9 and 5 μm) powders that were milled for 3, 6, 12, 18 and 24 h, respectively.

During the ball milling process, there are two competing processes: one is cold working of the powders which should lead to a decrease in ductility and eventual fracturing of the particles, while the other is cold welding of particles which tends to increase the particle size [14]. Figure 5 presents the histogram of the size distribution of the CNT/Al composite powders, in which (a) to (e), (f) to (j) and (k) to (o) are CNT/Al mixtures with the Al powder sizes of 20, 9 and 5 μm, corresponding to milling times of 3, 6, 12, 18 and 24 h, respectively. The average powder size increased slightly with the ball milling time for CNT/Al mixtures with Al powder size of 20 μm. The results indicate that particle welding may be a little more pronounced than the fracturing. For CNT/Al mixtures with Al powder sizes of 9 and 5 μm, however, the average powder size slightly decreased when the ball milling time was less than 6 h, and then slightly increased to some extent. The results indicate that equilibrium is achieved between particle welding and fracturing during the ball milling [22]. Unlike Esawi’s work [23], in this study, the ball milled particle size of the composite powder almost remains the same as the initial particle size except for some changes in morphology.

**Figure 6:**
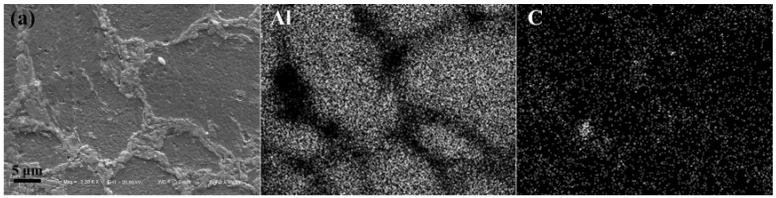

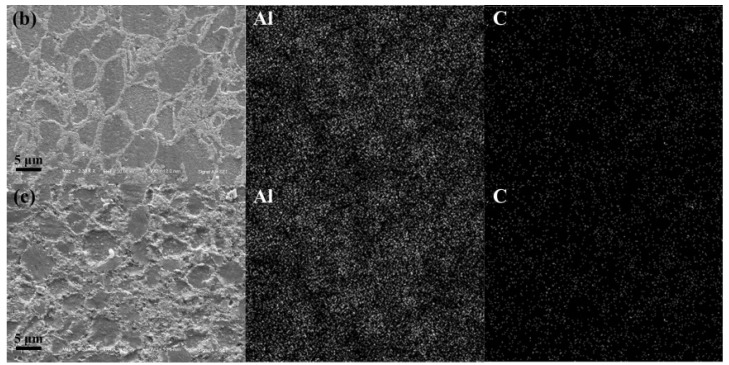
SEM-EDS analysis of the sintered composites that were ball milled for 24 h with different power sizes. (**a**) 20 μm; (**b**) 9 μm; (**c**) 5 μm.

Figure 6 gives the SEM image of the polished surface of the composites. Mapping of element Al and C was presented as well. For the composites of CNTs/Al (with the average powder size of 20 μm), CNTs clusters were observed at the grain boundary (Figure 6a), while for the other two composites that were milled for 24 h, CNTs were found to be uniformly dispersed in the matrix (Figure 6b,c).

The porosities of all the sintered composites are listed in Table 2. Overall, the porosities of all the composites first decrease with the increase of ball milling time, which results from the uniform dispersion of the CNTs, and then increases with further milling, which might be attributed to the occurrence of agglomeration in the powders at longer milling times as a result of the presence of very fine particles and the cold welding effect [29].

**Figure 7:**
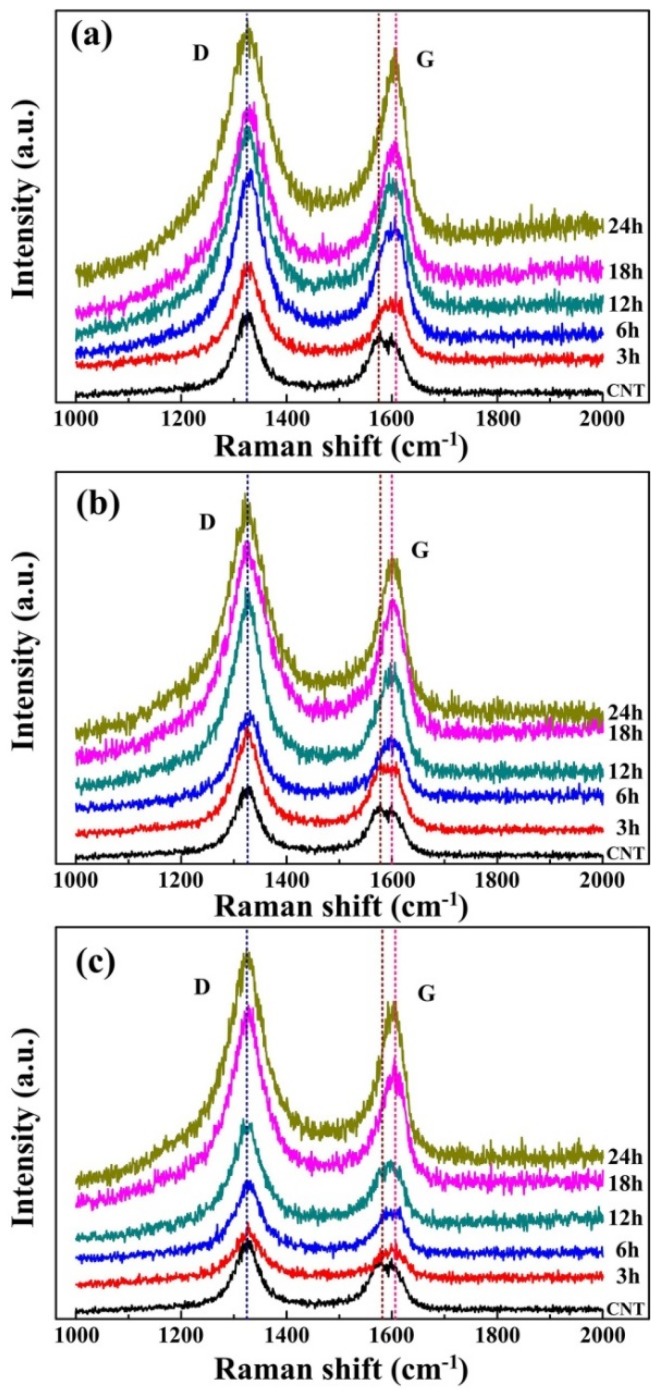
Raman spectra of initial CNTs and the composite powder varied according to milling time: (**a**) CNT and CNT ball milled with 20 μm Al; (**b**) CNT and CNT ball milled with 9 μm Al; (**c**) CNT and CNT ball milled with 5 μm Al.

The Raman technique is a powerful non-destructive method for revealing structural details [30]. Raman spectroscopy was usually used to assess the damage of the CNTs [31,32]. Figure 7 gives the raman spectrums of the milled composite powders. There are two important bands of raman spectrum, *i.e.*, the D band (at about 1350 cm^−1^) and the G band (at about 1580 cm^−1^) [15,33]. The D band corresponds to the A_1g_ breathing mode of sp^3^-hybridized carbon atoms of defects or amorphous carbon atoms in CNTs, while the G band corresponds to E_2g_ tangential mode of sp^2^-hybridized carbon atoms. The intensity ratio of the D band and the G band (ID/IG) is often used to assess the quality of the internal CNTs [12,18,21,34]. Figure 5 gives the raman spectrum of the milled composites. The peak shift of the G band from 1575 cm^−1^ to about 1600 cm^−1^ as shown in Figure 5a–c mainly arises from the infiltration of Al atoms in CNTs, causing slight distortion of sp^2^ bonding structure of CNTs, as revealed in Ref. [13,35].

**Figure 8:**
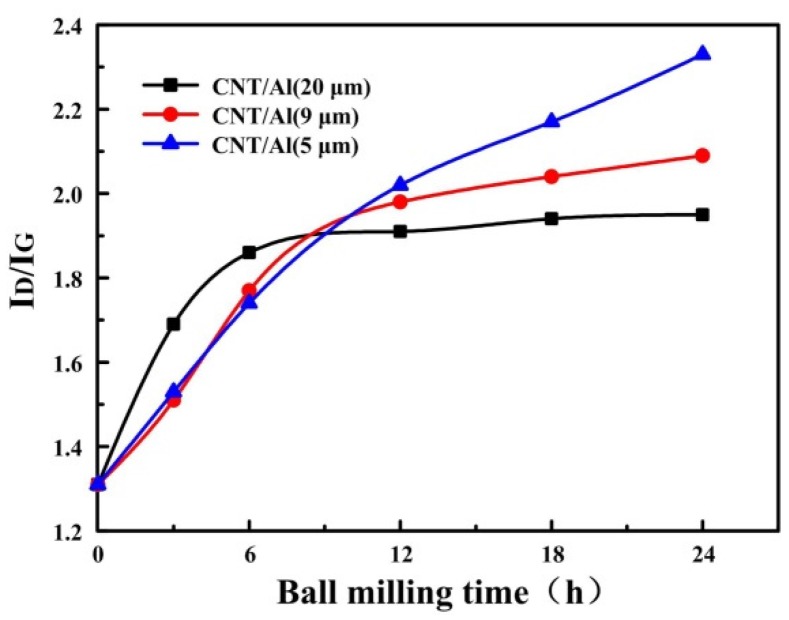
ID/IG ratio of the milled composites powders various with ball milling time.

Considering the fluctuations in the measurements, which appear frequently, separate changes in each peak could be hardly identified. Thus, we discuss the changes in ID/IG to evaluate the defects during ball milling. This is qualitative rather than quantitative. The ID/IG ratio of the three composite powders with different starting aluminum particles were calculated using the ratio of the integral intensity of the D band and the G band [36]. The result was plotted in Figure 8. It can be seen that the ID/IG ratio is on a rise after milling. For CNTs in the ball milled CNT/Al (with powder size of 20 μm) mixture, the ID/IG ratio first increased with the ball milling time increasing to 6 h, and then reached a plateau with further increases in the ball milling time. This results from the protective effect of the soft matrix [22,23]. It can be obviously seen from Figure 2 that most of the CNTs are embedded in the aluminum powder after 6 h of milling, which could protect the CNTs from damage during the milling. For CNTs in the ball milled CNT/Al (with powder size of 9 μm) mixture, the plateau occurs at the ball milling time of 12 h. This is consistent with the observation that most of the CNTs are embedded in the aluminum powder after 12 h of milling, as shown in Figure 4. While for CNTs in the ball milled CNT/Al (with powder size of 5 μm) mixture, the ID/IG ratio continues to climb from 1.31 to 2.33 with increases in the ball milling time. This indicates the continuous damage that is being made to the CNTs during the milling.

During the milling process, the kinetic energy at the moment of ball to powder collision would be converged as the strain energy of the powder. According to Ref. [13,14], the maximum dynamic deflection (δ_max_) is in proportion to D^−½^, and the maximum dynamic stress (δ_max_) is in proportion to D−32, where *D* is the diameter of powder. Although the proportional relationship is based on many assumptions and, hence, the proportion would be a lot different from the real ones, the significant difference could be induced for the different sizes of aluminum powder during milling. For the CNT/Al (with powder size of 5 μm) composite powder, the dynamic deflection (δ_max_) and the maximum dynamic stress (δ_max_) were much larger than the other two sizes of the aluminum alloy powders that are produced by the collision. So, the protective effect of the matrix might be significantly weakened due to the small size of the aluminum alloy particles.

**Figure 9:**
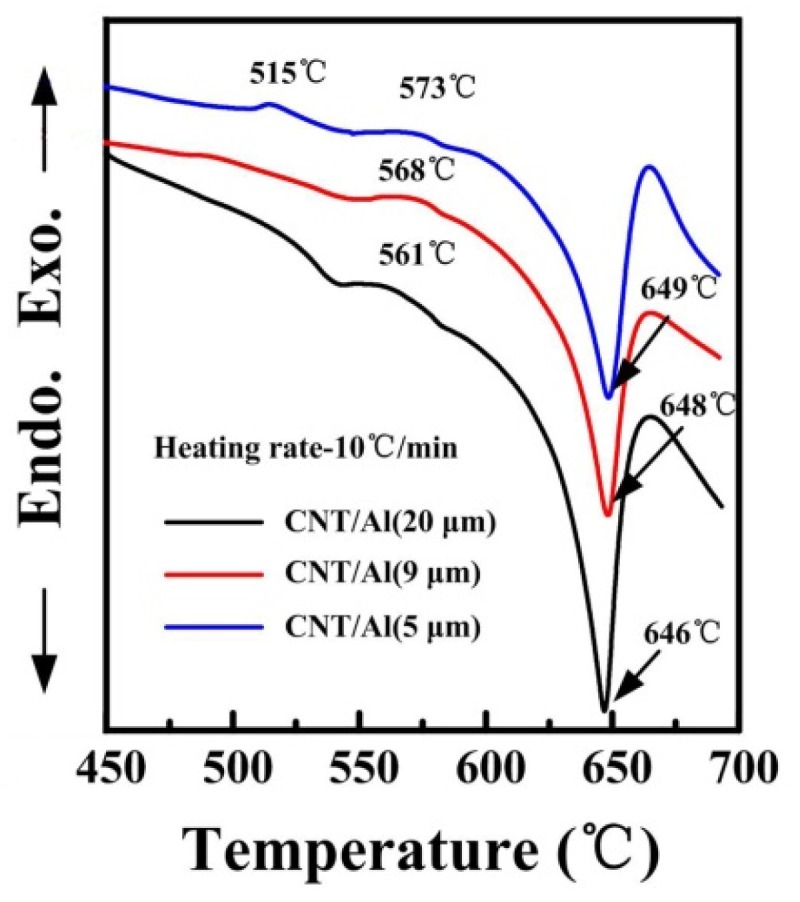
DSC plots of CNT/Al (with powder size of 20 μm), CNT/Al (with powder size of 9 μm) and CNT/Al (with powder size of 5 μm) composite powders that were ball milled for 24 h.

As previously reported [25], the reaction between damaged CNTs and Al matrix could occur at a lower temperature than the melting point of Al. The formation of the aluminum carbide (Al_4_C_3_) phase has a significant influence on the mechanical properties of the composites. Limited generation of Al_4_C_3_ at the interface is beneficial to the stress transfer from the matrix to the CNTs [11]. However, the excessive formation of the Al_4_C_3_ phase leads to a decrease of strength levels of the composites due to the carbides which have inferior properties compared to CNTs. To investigate the chemical stability of the damaged CNTs after ball milling, the DSC measurements were carried out at the heating rate of 10 °C/min. Figure 9 shows the DSC curves of the three composite powders with the aluminum matrix powder of 20, 9 and 5 μm, respectively, after a long milling time of 24 h.

As can be seen, the endothermic peaks of the three composite powders attributed to the melting of aluminum matrix is quite similar to each other [37,38]. While the position of the exothermic peak corresponding to the formation of Al_4_C_3_ phase occurs at 561 °C, 568 °C and 573 °C for aluminum powder with a powder size of 20, 9 and 5 μm, respectively, before the melting of the aluminum. The formation of Al_4_C_3_ before melting may be caused by the destruction of nanotubes during milling as observed by Poirier *et al.* [21] and Nayan *et al.* [25]. Note that there is an exothermic peak at 515 °C only appearing on the DSC curve of the CNT/Al (with powder size of 5 μm) composite powder. As previously reported, Al_4_C_3_ will start forming at around 450 °C after milling [21].

**Figure 10:**
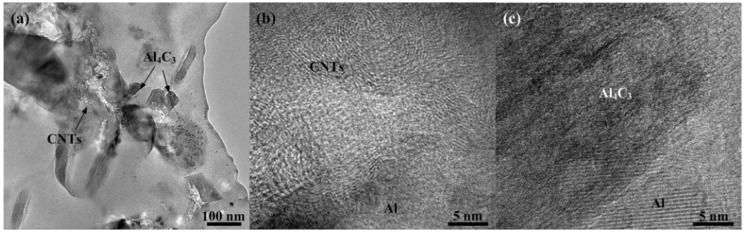
TEM images of the sintered CNT/Al (with powder size of 5 μm) composites that were ball milled for 24 h: (**a**) shows dispersion of the CNTs and the formation of Al_4_C_3_; (**b**) HRTEM image of CNTs in the composites; (**c**) HRTEM image of the Al_4_C_3_.

To determine the reaction at this temperature, the TEM experiments were performed on the composites with the aluminum matrix powder of 5 μm that milled for 24 h. Figure 10 presents TEM images of the sintered CNT/Al (with powder size of 5 μm) composites that were ball milled for 24 h. It can be seen that CNTs are randomly dispersed in the matrix and some Al_4_C_3_ are observed near the CNTs, as shown in Figure 10a. HRTEM of the CNTs and Al_4_C_3_ are shown in Figure 10b,c, respectively. The results indicate that after ball milling of 24 h and sintering, the CNTs still exist in the matrix. The lower reaction temperature of Al_4_C_3_ indicates the relative chemical instability of the CNTs in the 5 μm Al powder. It is known that the stability of CNTs reduces the damage to their structure imposed by the milling process. This possibly increases the CNT amorphization and supersaturated solid solution of carbon [29]. The results correspond to the level of damage of the CNTs in 5 μm Al powder, as shown in Figure 8. It is therefore believed that the milling of the CNT/Al (with powder size of 5 μm) will lead to more serious damage to the carbon nanotubes. They either form amorphous carbon clusters or dissolve into the Al matrix. Upon heating, the carbon atoms in solution transform into Al_4_C_3_ releasing heat [21].

**Figure 11:**
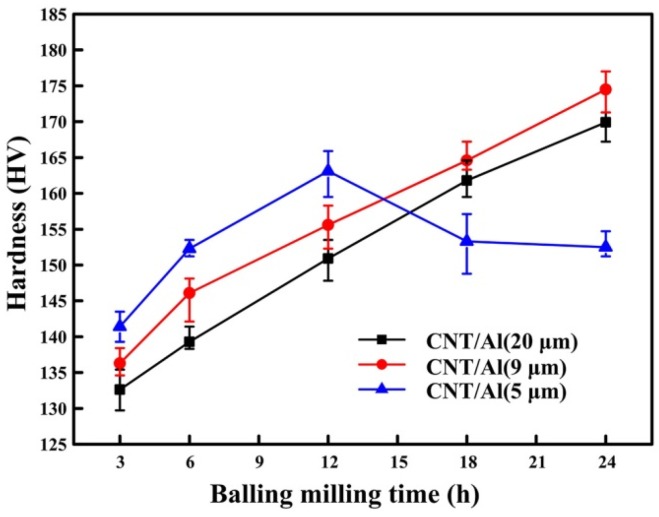
The effect on micro-hardness of the sintered composites for increasing ball-milling time and different initial aluminum alloy particle sizes.

Figure 11 presents the micro-hardness values of the hot pressed composites with the initial particle sizes of 20, 9 and 5 μm, respectively. The micro-hardness (HV) of the CNT/Al (with powder size of 20 μm) and CNT/Al (with powder size of 9 μm) composites gradually increases with the milling time rising from 131 to 170 and from 132 to 175 with the increase of ball milling time from 3 to 24 h, respectively. However, for the CNT/Al (with powder size of 5 μm) composite, the hardness firstly increases to a maximum hardness value of 163 with ball milling time. Subsequently, a decrease in hardness is observed for further milling. In general, the smaller the initial powder size, the higher the hardness for the composites that are ball milled for a certain time up until 12 h. For further milling up until 24 h, the trend was maintained for the composites prepared by 20 and 9 μm Al except for the 5 μm Al. Porosity has been considered to be a critical factor in the mechanical properties of materials, particularly in powder metallurgy. In this study, the composites prepared by Al (with powder sizes of 20, 9 and 5 μm) that were ball milled for a certain time possess similar porosities (in Table 2), and, hence, the effect of porosity on mechanical properties can be negligible. Therefore, the increase in hardness of the composites was attributed to the uniform dispersion of CNTs in the matrix with increasing ball milling time. The CNTs milled with Al (with powder size of 5 μm) for more time might be partly disintegrated into amorphous carbon [13], which is more likely react with the aluminum matrix [24], and the formation of Al_4_C_3_ is indicated in Figure 10.

The compressive strength of the composites that possess the highest micro-hardness of the three categories and the sintered CNT/Al (with Al powder size of 5 μm) that was ball milled for 24 h is listed in Table 3. The compressive stress was 613, 841 and 800 MPa for the composites prepared by Al powder with the powder sizes of 20, 9 and 5 μm that were ball milled for 24 h, respectively. Likewise, the fracture strain demonstrated an inverse trend to the size of the powder, which is 7.3%, 13.7% and 22.4%, corresponding to Al powders with the average sizes of 20, 9 and 5 μm, respectively, while the composites prepared with the Al powder size of 20 μm that were ball milled for 12 h exhibit a strength of 738 MPa and a fracture strain of 16.7%.

It has been reported [5,39] that some of the main factors responsible for enhancing the mechanical properties are the grain size, necessary dislocations and Orowan’s strengthening. In general, composite strength is improved by the addition of solid particles in the matrix. In this study, the content of CNTs in the composites was fixed (2 wt %). However, the distribution of CNTs in composites was different. As mentioned before, the clusters of CNTs can be observed at the grain boundary in the composites prepared by Al with a powder size of 20 μm, while CNTs in the other two composites prepared by Al with powder sizes of 9 and 5 μm exhibit uniform distribution. The clusters of CNTs are detrimental to the overall mechanical properties, and thus, the composites with Al powder sizes of 9 and 5 μm show better mechanical properties. The formation of Al_4_C_3_ is due to the reaction of aluminum matrix and the seriously damaged CNTs, leading to the consumption of CNTs. Therefore, the formation of the Al_4_C_3_ phase leads to a decrease of strength of the composites because Al_4_C_3_ has lower hardness than CNTs.

## 4. Conclusions

In this study, the effect of the initial 2014 aluminum alloy particle size on CNTs, in terms of damage, during the ball milling process was investigated. The following conclusions can be drawn:

1. With the decrease in the size of the aluminum alloy particles, the ball milling time needed for CNTs to fully disperse into the aluminum matrix significantly increases. In particular, the CNTs are still observed on the surface of the aluminum particle after 24 h of milling when the average aluminum particle size is 5 μm.
2. For CNTs in the ball milled CNT/Al (with powder size of 20 μm) and CNT/Al (with powder size of 9 μm) mixture, the intensity ratio of the D band and the G band (ID/IG) first increased and then reached a plateau with the ball milling time increasing. This is mainly due to the fact that most of the CNTs are embedded in the aluminum powder after milling to a given extent, which could protect the CNTs from damage during the milling; while for CNTs in the ball milled CNT/Al (with powder size of 5 μm) mixture, the ID/IG ratio continues to climb from 1.31 to 2.33 with increases in the ball milling time. This indicates that continuous damage occurs in the CNTs during milling.
3. The more serious the damage to the CNTs is, the greater their chemical instability, and can result in the formation of Al_4_C_3_ at a lower temperature before the melting of aluminum. For a given weight percentage of CNTs (2.0 wt %), the formation of the Al_4_C_3_ phase leads to the consumption of CNTs, thus resulting in a decrease in micro-hardness and compressive strength of the composites, as the carbides have inferior properties compared to CNTs.

## Figures and Tables

**Table 1:** Chemical compositions of the 2014 Al matrix (wt %).

Element	Cu	Si	Mg	Mn	Fe	Al
compositions	4.8	1.2	0.8	1.0	0.7	Balance

**Table 2:** Porosities of the Sintered Composites (%).

Average Powder Size (μm)	Ball Milling Time (h)				
	3	6	12	18	24
20	3.7	2.0	3.3	3.7	4.4
9	3.3	3.3	2.0	2.9	4.3
5	3.7	1.5	2.2	3.7	4.7

**Table 3:** Compressive strength and fracture strain of the sintered composites.

Average Al Powder Size (μm)	Ball Milling Time (h)	Compressive Stress (MPa)	Fractures Strain (%)
20	24	613 ± 18	7.3 ± 0.3
9	24	841 ± 2	13.7 ± 0.1
5	24	800 ± 8	22.4 ± 0.2
5	12	738 ± 2	16.9 ± 0.2
